# Type of Recurrence, Cause of Death and Second Neoplasms among 737 Patients with Ductal Carcinoma In Situ of the Breast—15-Year Follow-Up

**DOI:** 10.3390/cancers14030669

**Published:** 2022-01-28

**Authors:** Anna Niwińska, Michał Kunkiel

**Affiliations:** Department of Breast Cancer and Reconstructive Surgery, Maria Sklodowska-Curie National Research Institute of Oncology, Roentgen 5 Str., 02-784 Warsaw, Poland; michal.kunkiel@gmail.com

**Keywords:** DCIS, ductal carcinoma in situ, true recurrence, second malignancy, contralateral breast cancer, cause of death, long-term outcomes, de-escalating treatment

## Abstract

**Simple Summary:**

A retrospective analysis of 737 consecutive DCIS patients with a 15-year follow-up was carried out. Sixty-six recurrences (42% DCIS, 58% invasive) were reported: 61 in the breast and 5 outside the breast. 79% of local recurrences were true recurrences. The highest number of recurrences was reported in patients after local excision without radiotherapy despite the fact that it was the lowest-risk group. Deaths due to DCIS progression were reported in 0.5% of all patients and in 10.5% of patients with invasive recurrences. The majority of deaths were linked to the age of the patients or other diseases, including other neoplasms.

**Abstract:**

**Aim:** To assess the outcomes of 737 consecutive patients with DCIS, with particular attention to the type of recurrences, other malignancies and causes of deaths. **Material and Methods:** A retrospective analysis of 737 consecutive DCIS patients treated in one institution in the years 1996–2011 was carried out. The cumulative recurrence risk, DFS, OS depending on the method of treatment (mastectomy, breast-conserving treatment (BCT), breast-conserving surgery (BCS)) and cause of death were assessed. **Results:** Sixty-six recurrences (42% DCIS, 58% invasive) were reported: 61 in the breast and 5 outside the breast. The cumulative recurrence risk after a 15-year observation after mastectomy, BCT and BCS was 3.2%, 19.5% and 31.2%, respectively (*p* < 0.001). The 15-year DFS after mastectomy, BCT and BCS was 72%, 65% and 48%, respectively (*p* < 0.001). The 15-year OS after mastectomy, BCT and BCS was 75%, 83% and 70%, respectively (*p* = 0.329). Deaths due to DCIS progression were reported in four (0.5%) of the overall patients and in 10.5% of patients with invasive recurrences. The majority of deaths were linked to the age of the patients or other diseases, including other neoplasms, but not DCIS. **Conclusions:** The highest number of recurrences was reported in patients after BCS, despite the fact that it was the lowest-risk group. In total, 79% of local recurrences were true recurrences and 58% were invasive recurrences. Local recurrences were effectively treated without an influence on the OS. The percentage of deaths due to DCIS was low and mainly concerned patients with locoregional and distant failure.

## 1. Introduction

Ductal carcinoma in situ (DCIS) is still a challenge for oncologists for several reasons. Firstly, due to the commonness of mammographic screening, DCIS has come to constitute approximately 20–25% of breast cancers detected with this method, so it has emerged as an epidemiological problem [1,2,3]. Secondly, DCIS covers a broad spectrum of changes and has a distinct and often unpredictable course [1,4,5,6]. Among the methods of treatment, breast-conserving surgery (BCS, local excision without radiation therapy), breast-conserving treatment (BCT, local excision plus radiation therapy), mastectomy and endocrine therapy are used. However, there is still a problem with selecting patients with a low risk of local recurrence in whom radiation therapy after local excision can be safely avoided [7,8]. Thirdly, in patients with local recurrence, it is difficult to distinguish between a true local recurrence and another primary ipsilateral breast cancer [9,10]. This knowledge should have an influence on the primary treatment of DCIS. Finally, recurrence in the form of pure DCIS is not a relevant clinical problem because this type of relapse does not influence survival. The question of how many patients with invasive recurrence die from the dissemination of the disease seems to be more important [11,12]. Knowledge of the risk and frequency of invasive recurrence could help to establish the proper choice of initial treatment of DCIS.

## 2. Aim

The aim of the study was to assess the results of the treatment of 737 consecutive DCIS patients treated in one institution in the years 1996–2011, with particular attention to the type of recurrences (true recurrences vs. second primary ipsilateral breast cancer; invasive recurrence vs. pure DCIS recurrence), other malignancies and causes of death.

## 3. Patients and Methods

### 3.1. Patients

The study was retrospective. The database included 737 consecutive cases of pure DCIS patients treated in the National Research Institute of Oncology in Warsaw in the years 1996–2011. All DCIS patients with microinvasion were excluded from the study. Mastectomy, BCT (breast-conserving treatment, local excision plus radiation therapy) and BCS (breast-conserving surgery, local excision without radiation therapy) were performed based on risk factors and/or the patient’s preference. From 2004, patients were treated on the basis of the VNPI prognostic index of 2003, developed on the basis of 4 risk factors [13]. Over the 16 years covered in the study, radiation therapy was performed with Co 60 gamma rays of 1.25 MV, or 4 MV photons or 6 MV photons, with a fraction dose of 2–2.5 Gy to a total dose of 42.5–50 Gy. A boost of 5 fractions of 2 Gy to a total dose of 10 Gy was applied in 58 (24%) patients with unfavorable prognostic factors. None of the patients in the total group were treated with adjuvant hormone therapy with tamoxifen.

All the patients routinely reported for follow-up checks every 6 months during the first 2 years after the completion of the treatment and every year thereafter. Data concerning the condition of the patients were systematically recorded in their case histories. Mammography was performed every 6 months during the first 2 years after the treatment and then annually. The time of observation for the whole group was 6–21 years; the median time of observation (95% confidence interval) was 120 months (114–126 months).

### 3.2. Methods

A descriptive analysis of treatment failures was performed. In order to distinguish true local recurrences from other primary breast cancers in the treated breast, mammographic pictures from the initial diagnosis of DCIS prior to the primary treatment were compared with mammography after the detection of the recurrence. Based on the literature, recurrences in the tumor bed or in the same quadrant of the breast, at a distance of 5 cm maximum from the initial lesion described on the primary mammography, were assumed to be true local recurrences. All other recurrences in the treated breast were assessed as other primary breast cancers [9,10].

In order to compare the biological subtype of the initial DCIS and the recurrence, an analysis of estrogen receptor (ER), progesterone receptor (PR), human epidermal growth factor receptor 2 (HER2) and Ki-67 protein was performed. The method of competing hazards was used to assess the disease-free survival (DFS), the risk of recurrences and the overall survival (OS) in the entire group and in the subgroups treated with mastectomy, BCT and BCS. The Cox proportional hazard model was used to assess the prognostic value of recurrence risk factors in 400 patients treated with breast conservation (BCS + BCT).

The rates and types of other neoplasms detected before, concurrently with or after DCIS diagnosis were analyzed. The rate of deaths due to DCIS progression, other neoplastic tumors and other causes was calculated.

### 3.3. Statistical Analysis

The DFS was measured from the date of the surgery to the first failure or, in the absence of treatment failure, the date of the last clinical observation. The cumulative percentage of recurrences was calculated with the competing hazards methods, with death for other reasons as the competing hazard. The OS was measured from the surgery to death or the date of the last information that the patient was alive. The Kaplan–Meier method was used to calculate the likelihood of survival.

For the group treated with breast conservation, an analysis was conducted of 6 prognostic factors for the risk of failure using the Cox proportional hazard method. The modeling process involved the use of the stepwise variables elimination method with inclusion and exclusion criteria of 0.05 and 0.1, respectively. All the estimates are given with a 95% confidence interval. Critical test values lower than 0.05 were considered statistically significant. The analysis was performed using the IBM SPSS 23.0.0.2 statistical packet and R version 3.4.4.

## 4. Results

Among the 737 patients, 337 (46%) underwent mastectomy, 241 (33%) underwent BCT and 159 (23%) underwent BCS. Breast reconstruction was performed in 64 patients after mastectomy. Out of 400 patients who received breast-conserving surgery, with or without radiotherapy, 104 (26%) required at least one re-excision for negative margins. The clinical characteristics of the whole group, with subdivision according to the method of treatment, are given in Table 1.

### 4.1. Frequency and Type of Recurrence

In the median observation time of 120 months, DCIS recurrence was reported in 66 (9%) patients. In 61 patients, it was local recurrence in the treated breast; in four, it was recurrence in regional lymph nodes, while in one patient, there were metastases to the liver and bones. In 42% of the cases, the recurrence had the form of a pure DCIS, while in 58%, it had the form of an invasive cancer or a mixed form of DCIS with an invasive breast cancer component.

The comparison of mammography images before the initial treatment and after local recurrence revealed true recurrence in the breast in 48/61 (79%) cases. In 21% of cases, the recurrence took place in another breast quadrant, which gives rise to suspicion of another cancer in the same breast. Table 2 presents the characteristics of the 66 recurrences in the group of 737 patients, and Table 3 presents the characteristics of the 66 recurrences with subdivision according to the method of initial treatment.

In the 66 patients with failure, the number of triple-negative (ER−PR−HER2−), luminal HER2-positive (ER/PR+HER2+), non-luminal HER2-positive (ER−PR−HER2+), luminal A (ER+PR+HER2−Ki-67 ≤ 20%) and luminal B (ER+PR+HER2−Ki-67 > 20%) subtypes in initial DCIS was 4, 7, 12, 35 and 8, respectively. After recurrence, the rate of these biological types was 5, 7, 12, 31 and 11, respectively.

### 4.2. Cumulative Recurrence Risk

**Figure 1:**
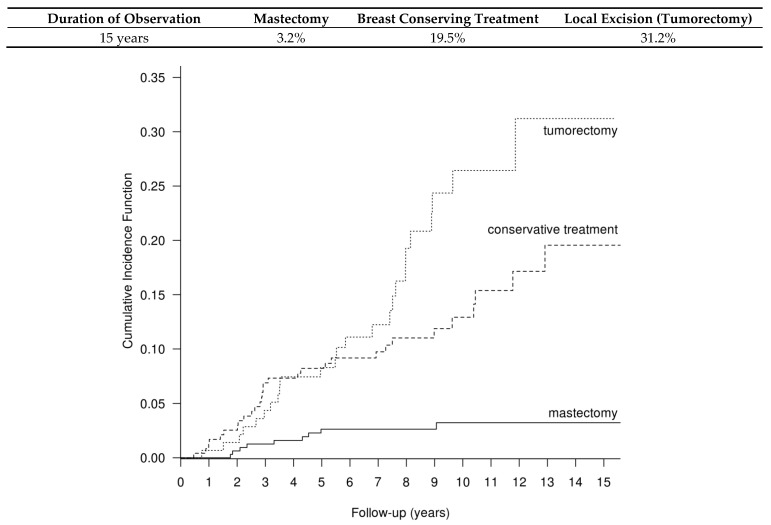
Cumulative recurrence risk after mastectomy, BCT and BCS in the group of 737 DCIS patients, calculated with the method of competitive hazards; *p* < 0.001.

The competing risk of recurrences after mastectomy, BCT and BCS is presented in Figure 1. The 5-year cumulative recurrence risk after mastectomy, BCT and BCS was 2.6%, 8% and 8%; the 10-year cumulative recurrence risk was 3.2%, 13% and 26.4%; and the 15-year recurrence risk was 3.2%, 19.55% and 31.2%, respectively (*p* < 0.001).

### 4.3. Disease-Free Survival (DFS)

**Figure 2:**
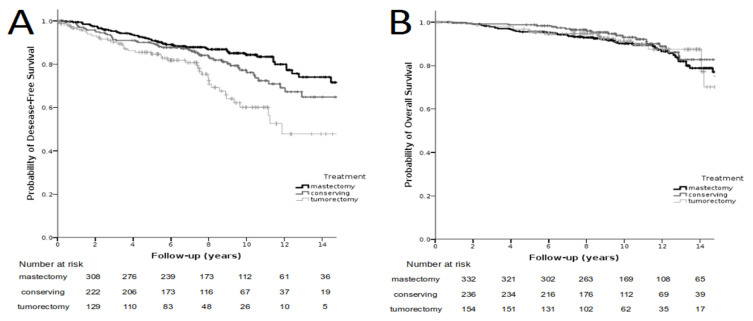
Recurrence-free survival in the group of 737 patients depending on the type of treatment; *p* < 0.001 (left side, (**A**)). Overall survival in the group of 737 patients depending on the method of treatment; *p* = 0.329 (right side, (**B**)).

In the whole group of 737 patients, the 5-, 10-, 15- and 20-year DFS amounted to 89%, 77%, 65% and 59%, respectively. Figure 2A gives the DFS in the group of 737 patients, with subdivision according to the method of treatment. The 5-year DFS after mastectomy, BCT and BCS amounted to 91%, 90% and 85%, respectively. The 10-year DFS after mastectomy, BCT and BCS amounted to 84%, 76% and 60%, respectively, and the 15-year DFS after the respective treatments amounted to 72%, 65% and 48% (*p* < 0.001).

### 4.4. Overall Survival (OS)

In the entire group, the 5-, 10-, 15- and 20-year overall survival amounted to 97%, 91%, 77% and 70%, respectively. Figure 2B presents the OS in the group of 737 patients with subdivision according to the method of treatment. The 5-year OS after mastectomy, BCT and BCS amounted to 96%, 99% and 96%, respectively. The OS after 10-year observation following mastectomy, BCT and BCS amounted to 90%, 93% and 91%, respectively. The 15-year OS amounted to 75%, 83% and 70%, respectively, and did not statistically significantly differ depending on the treatment method (*p* = 0.329).

### 4.5. Cox Analysis

Cox analysis of the recurrence function in the group of 400 patients treated with breast-conserving surgery with or without radiotherapy (BCT + BCS) was carried out. The value of the following prognostic factors was analyzed: patient age, method of DCIS diagnosis (mammography alone vs. clinically explicit), DCIS size, nuclear grade (NG), presence of comedo necrosis and size of the margin around the excised DCIS. None of the analyzed factors were found to have any statistically significant influence on the risk of recurrence. The *p*-value for all the risk factors in both analyses was *p* > 0.1.

### 4.6. Other Neoplastic Lesions

In the course of the whole observation period, in the group of 737 DCIS patients, 124 other neoplastic lesions were reported in 121 patients (16%), including 57 (8%) contralateral breast cancers, 15 endometrial cancers, 8 lung cancers, 6 thyroid cancers, 5 melanomas, 4 ovarian neoplasms, 4 cervical cancers, 3 skin cancers, 3 gallbladder and/or liver hilum cancers, 2 colon cancers, 2 urinary bladder cancers, 2 gastric cancers, 2 pancreatic cancers, 2 head and neck cancers, 2 cancers of unknown site, 2 sarcomas, 1 kidney cancer, 1 salivary gland cancer, 1 vaginal cancer, 1 glioblastoma multiforme and 1 meningioma. Out of the 58 patients with a contralateral breast cancer, it was detected prior to the DCIS diagnosis in 13, simultaneously with DCIS in 3 and after the diagnosis of DCIS in 42 patients.

### 4.7. Causes of Deaths

In the course of the whole observation of 737 patients (6–21 years, median 10 years), 86 deaths were recorded. The causes of the deaths are presented in Table 4. In 26 patients, death was due to a malignancy other than breast cancer. In 48 patients, death occurred for other reasons. Deaths due to other reasons were deaths confirmed by hospital documentation after hospitalization for non-oncological diseases as well as by check-ups in our institution in the year prior to death that did not reveal a DCIS recurrence or progression of other neoplasms. Most of those patients were above 80 years of age.

### 4.8. Characteristics of Four Patients Who Died Due to DCIS Recurrence

DCIS recurrence was the cause of death only in four cases, which accounted for 0.5% of the 737 patients. In all the four patients who died, an invasive breast cancer recurrence was detected. The age at initial diagnosis of those patients was 48, 57, 62 and 65 years. Mastectomy was performed in three patients and BCT in one patient as the initial DCIS treatment. The dimensions of the DCIS in the four patients were 1.5 cm; 2 cm, with microcalcifications in the area of 5 cm; 0.9 cm and 3 cm. The narrowest free margins were 0.1 mm, 3 mm, 9 mm and 10 mm. Nuclear grade NG3 was assessed in three patients and NG1 in one patient. Out of the four patients, an invasive local recurrence in the breast with lymph nodes involvement was detected in one, metastases to regional lymph nodes were detected in two and in distant metastases were detected in one. The time to the recurrence differed depending on the biological subtype: in the two patients with the luminal A subtype (ER/PR+HER2−Ki-67 ≤ 20%), it was 66 and 108 months, while in the two patients with HER2-positive breast cancer, it was 19 and 29 months. Other cancers were not detected before and after the recurrence in any of the four patients who died.

## 5. Discussion

### 5.1. Type of Recurrence

In the present study, 66 recurrences were disclosed in the group of 737 consecutive patients with DCIS treated without any selection by different options and with a long follow-up time. In 61 patients, the recurrence was located in the breast; in four patients, it was located in regional lymph nodes; and in one—in parenchymal organs. Among the 61 patients with local recurrence, 79% of recurrences were localized in the tumor bed or in the same quadrant of the breast up to 5 cm from the location of the primary lesion and were considered to be true recurrences. In 21% of those cases, the recurrence occurred in another quadrant of the treated breast, and this was treated as second primary ipsilateral breast cancer. In the few studies concerning the percentage of true recurrences and another breast cancer after the initial DCIS diagnosis, the percentage of true recurrences was lower than in our study and amounted to 69% and 66%, respectively [10,14].

In the present study, the percentage of local recurrences in the form of a pure invasive breast cancer or an invasive breast cancer with a DCIS component amounted to 54%. In the remaining 46%, local recurrences were classified as pure DCIS. This is consistent with the results of numerous studies in which the percentage of recurrences in the form of an invasive cancer was 48–76% [10,15,16,17].

Although neoplastic cells do not extend outside the basal membrane in DCIS, distant metastases are observed in approximately 0.5% of cases [18]. They are likely to result from a microinvasion of cancer cells unrecognized on histopathological examination after the primary surgical procedure or from recurrence in the form of an invasive cancer. In the present study, metastases to regional lymph nodes were detected in four patients and distant metastases in one patient. It is hard to explain this phenomenon, especially since the presented material included 737 DCIS patients without microinvasion. Our pathologist performed repeated histopathological examination of paraffin blocks from the initial DCIS of patients to detect microinvasion, but no cancer invasion foci were disclosed.

### 5.2. Relationship between Type of Treatment and Risk of DCIS Recurrence

The outcomes of the 737 DCIS patients clearly show that mastectomy remains the most effective method of treatment as regards the risk of recurrence. In 337 patients who underwent mastectomy, the percentage of failures after 15 years of observation was 2.6%. According to the literature, mastectomy allows to obtain 98–99% permanent recoveries, with the percentage of recurrences after 5–10 years amounting to 1–2.6% [19,20,21] and that of invasive recurrences amounting to 0.5% after 12 years of observation [22,23].

In the analyzed study, in 241 patients after BCT, the percentage of failures after 15 years of observation was 19.5%. The role of radiation therapy was confirmed in four clinical studies with randomization [11,12,24,25]. The studies confirmed that radiation therapy halves the risk of local recurrence both in the form of an invasive cancer and DCIS. Three of the meta-analyses referred to above indicated that radiation therapy after BCS caused a reduction in the relative recurrence risk by 50% but had no influence on survival [26,27,28].

In the present study, the percentage of recurrences in 159 patients after BCS (without radiotherapy) on 15-year observation was 31.2%. In four randomized clinical trials and a meta-analysis, the percentage of recurrences after local excision without radiation therapy after 12–15 years of observation was 19–35% [11,12,24,25]. In the meta-analysis of Boyages, the risk of recurrence after mastectomy, BCT and BCS was 1.4%, 8.9% and 22.5%, respectively [20]. In a meta-analysis of 5 prospective and 21 retrospective studies with an observation time of at least 10 years, the total percentage of recurrences after mastectomy, BCT and BCS was 2.6%, 13.6% and 25.5%, respectively, while the percentage of recurrences in the form of an invasive breast cancer was 2.5%, 7.4% and 11%, respectively [19]. In some new articles, the rate of recurrence in patients with DCIS after BCS is much lower than that presented above, but the characteristics of the evaluated groups of patients were more favorable than in our study (DCIS < 2.5 cm, mammographically detected, NG1/G2 with margin ≥ 3 mm) [7,8,29].

Despite scientific evidence that radiation therapy reduces the risk of DCIS relapse, attempts at defining the low-risk group of patients who do not require radiation therapy after BCS have thus far failed. Two clinical trials were conducted in order to answer this question: ECOG E5194 [8] and RTOG 9804 [7]. In the E5194 study, the rate of local recurrences in patients without radiotherapy was 24.6% after 12 years of observation, and invasive recurrences accounted for more than half of the cases (13.4%) [8]. In the second study, after 10 years of observation, the rate of recurrences was 9.1% [7]. These data suggest that it is difficult to define a group that can safely avoid being treated with radiation therapy. It seems that there is a risk of recurrence in every group of DCIS patients, and this risk persists for many years after the initial diagnosis.

### 5.3. Cause of Deaths: Does Recurrence Mean Death?

In the analyzed material, deaths due to DCIS progression were reported in 0.5% (4/737) of all treated patients and an invasive recurrence in 10.5% (4/38) of patients. The results of the treatment after diagnosis of a local recurrence (recurrence in the breast) were very good, irrespective of whether it was invasive or non-invasive. By contrast, all four invasive recurrences in the form of lymph node metastases or distant metastases led to death despite intensive salvage treatment. Three of the four patients who died were initially treated with mastectomy due to extensive DCIS disease.

The results of the present study are consistent with the observations of other authors with regard to the type of recurrence and risk of death. A meta-analysis of four randomized trials showed that the risk of death due to breast cancer in patients with DCIS is low irrespective of the method of treatment [30]. In another meta-analysis, the percentages of deaths due to breast cancer after mastectomy, BCT and BCS after 10-year observation were comparable and amounted to 2%, 2.4% and 2.6%, respectively [19]. In patients with DCIS treated with BCT, the cumulative risk of breast-cancer-specific mortality after 10, 20 and 30 years of observation was 0.8–2%, 3.3% and 6.3%, respectively [31,32,33,34,35]. The risk of death in patients under 50 with DCIS is higher than in older ones (HR = 1.7) [36]. In particular, this refers to women under 35 years of age (8.8% and 3.2% after 20 years of observation, respectively; HR = 2.58; *p* < 0.001) [32,37].

In our material, we did not observe a correlation between age and the risk of recurrence or death, probably because young patients were treated by us as high-risk patients and radiation therapy after BCS or even mastectomy with reconstruction was applied in all of them. It may be why young age was not found to be a recurrence-influencing factor in the Cox analysis.

The low risk of death due to breast cancer in patients with DCIS should not diminish the vigilance of oncologists. While non-invasive recurrences can be treated effectively without an influence on survival, invasive recurrences require more intensive local and systemic treatment, worsen the quality of life and make prognosis uncertain. The NSABP B-17 and B-24 clinical trials revealed that DCIS recurrence in the form of invasive breast cancer increases the risk of death to 10.4% after 10 years of observation [11], which is consistent with our study where death was reported in 10.5% (4/38) of patients with an invasive recurrence. In the EORTC 10853 study, the 10-year breast-cancer-specific survival (BCSS) and the 10-year overall survival (OS) in patients with an invasive recurrence were much worse (BCSS HR = 17.66 and OS HR = 5.17) than in patients with recurrence in the form of pure DCIS or in patients without any recurrence. A reduction in BCSS from 95% to 60% after an invasive local recurrence was noted [12]. The results of those studies indicate the necessity of effective treatment of primary DCIS in order to avoid invasive recurrences worsening prognosis.

In our clinic, the type of treatment depended on risk factors: the low-risk group was treated with BCS, the medium-risk group with BCT and the high-risk group with mastectomy. The rate of recurrence after BCS (low-risk group) was the highest, but fortunately, it had no influence on survival. No patient initially treated with BCS died due to an invasive or non-invasive recurrence. It must be noted that all DCIS patients after BCS were subject to intensive follow-up in order to ensure very early detection of a potential recurrence on mammography. In contrast to the group treated with BCS, three of the four patients who died due to the recurrence were initially treated with mastectomy due to very extensive DCIS. In those patients, locoregional and distant metastases were detected, leading to death despite intensive salvage treatment.

### 5.4. Other Neoplastic Lesions

In the present study, the rate of contralateral breast cancer was 8%, which is in agreement with the literature. In two studies, contralateral breast cancer was detected in 5% [38] and 7.9% [29] of patients with DCIS after 10 years of observation.

### 5.5. Strengths and Limitations of the Study

What speaks in favor of the obtained results is the fact that DCIS patients remain under observation in our institution until the end of their life, which allows for more accurate assessment of their outcomes. In addition, the low percentage of deaths after DCIS recurrence is evidence of the modern and effective treatment provided in our institution, which is organized as a comprehensive cancer center.

The limitation of the study lies in its retrospective character. In addition, adjuvant endocrine therapy, which could have had an influence on the course of the disease, was not administered to any of the patients treated in the years 1996–2011.

## 6. Conclusions

1. The highest recurrence rate was observed in low-risk patients treated with BCS without radiation therapy (31% during 15 years of observation). However, those patients were treated effectively irrespective of the type of the local recurrence (invasive or non-invasive), without influence on survival. No death due to breast cancer was detected in this group.
2. The majority (79%) of local recurrences were true recurrences, localized in the tumor bed or up to 5 cm in the same quadrant. They did not differ from the initial DCIS in terms of the biological subtype. Of the recurrences, 58% were in the form of an invasive breast cancer or an invasive breast cancer with a DCIS component. Additionally, 10.5% (4/38) of patients with invasive breast cancer died.
3. The rate of death in the whole treated group was 0.5%. Local recurrences (in the breast) detected in the form of DCIS and in the form of an invasive breast cancer were treated effectively without an impact on the overall survival. Invasive recurrences in the form of lymph node metastases or distant metastases (four cases, 10.5% of invasive recurrences) led to death, despite intensive salvage treatment. In those four cases, the time from the initial diagnosis of DCIS to the detection of an invasive recurrence depended on the biological subtype of DCIS.

## Figures and Tables

**Table 1:** Characteristics of 737 consecutive patients with DCIS, with subdivision into the methods of treatment.

Feature	Total 737 Patients (%)	Mastectomy 337 Patients (%)	BCT * 241 Patients (%)	BCS ** 159 Patients (%)
Age (years)				
<40	29 (4)	19 (6)	7 (3)	3 (2)
40–50	142 (19)	64 (19)	49 (20)	29 (18)
51–60	293 (40)	123 (36)	110 (46)	60 (38)
>60	273 (37)	131 (39)	75 (31)	67 (42)
Method of DCIS diagnosis				
Mammography alone Clinically explicit	643 (87)	275 (82)	228 (95)	140 (88)
	94 (13)	62 (18)	13 (5)	19 (12)
DCIS size on mammography (cm)				
<1	246 (33)	73 (22)	96 (40)	77 (49)
1.1–2.5	249 (34)	78 (23)	104 (44)	67 (42)
2.6–4	123 (17)	84 (25)	26 (10)	13 (8)
>4	119 (16)	102 (30)	15 (6)	2 (1)
DCIS size on histopathological examination (cm)				
<1	278 (38)	58 (17)	98 (41)	122 (77)
1.1–2.5	238 (32)	89 (26)	117 (48)	32 (20)
2.6–4	97 (13)	70 (21)	22 (9)	5 (3)
>4	124 (17)	120 (36)	4 (2)	0
The narrowest margin (mm)				
<1	0	0	0	0
1–2	232 (32)	143 (43)	30 (12)	59 (37)
3–9	129 (17)	110 (33)	2 (1)	17 (11)
≥10	376 (51)	84 (25)	209 (87)	83 (52)
Histological malignancy grade				
NG1	151 (20)	40 (12)	33 (14)	78 (49)
NG2	296 (40)	155 (46)	89 (36)	53 (33)
NG3	290 (40)	142 (42)	119 (50)	28 (18)
Comedo necrosis				
Present	271 (37)	119 (35)	119 (49)	33 (21)
Absent	466 (63)	218 (65)	122 (51)	126 (79)

* BCT—breast-conserving therapy; ** BCS—breast-conserving surgery.

**Table 2:** Pooled characteristics of 66 recurrences in 737 DCIS patients.

Feature	Number of Patients with Recurrence (%)		
Character of recurrence			
Local recurrence	61/737 (8)		
Metastases to lymph nodes	4/737 (0.5)		
Distant metastases (liver and bones)	1/737 (0.1)		
Location of local recurrence			
Recurrence in the tumor bed	32/61 (53)	79% true recurrences	
Recurrence in the same quadrant	16/61 (26)		
Recurrence in another breast quadrant	13/61 (21)	21% likely another primary breast cancer	
Histological type of the recurrence			
DCIS	28/66 (42)		
DCIS + invasive cancer	31/66 (47)	58% recurrences in the form of invasive cancer	
Invasive cancer	7/66 (11)		
Biological type of the recurrence *			
ER−PR−HER2−	5/66 (8)		
ER−PR−HER2+	12/66 (18)		29% HER2+ cancers
ER/PR+HER2+	7/66 (11)		74% luminal cancers
Luminal A	30/66 (45)		63% luminal HER2-negative cancers
Luminal B	12/66 (18)		

* ER—estrogen receptor; PR—progesterone receptor; HER2—human epidermal receptor 2; Luminal A—ER/PR+HER2−Ki-67 ≤ 20%; Luminal B—ER/PR+HER2−Ki-67 > 20%.

**Table 3:** Pooled characteristics of 66 recurrences, with subdivision according to the method of treatment.

Feature	Mastectomy Number of pts (%)	BCT Number of pts (%)	BCS Number of pts (%)
Number of patients	337	241	159
Recurrences			
Yes	9 (2.6)	32 (13.2)	25 (15.7)
No	328 (97)	209 (87)	134 (84)
Histological type of the recurrence			
DCIS	0	15/32 (47)	11/25 (44)
DCIS + inv. cancer *	0	11/32 (34)	13/25 (52)
Invasive cancer	9/9 (100)	6/32 (19)	1/25 (4)
Biological type of the recurrence **			
ER−PR−HER2−	0	4/66 (6)	1/66 (2)
ER/PR-HER2+	4/66 (6)	6/66 (9)	2/66 (3)
ER/PR+HER2+	3/66 (4.5)	2/66 (3)	2/66 (3)
Luminal A	2/66 (3)	12/66 (18)	16/66 (24)
Luminal B	0	8/66 (12)	4/66 (6)
Recurrence location			
Tumor bed	5/9 (55)	16/32 (50)	14/25 (56)
The same quadrant	-	8/32 (25)	6/25 (24)
Another quadrant	-	7/32 (22)	5/25 (20)
Metastases to lymph nodes	3/9 (34)	1/32 (3)	0
Distant metastases	1/9 (11)	0	0

* DCIS + inv. cancer—recurrence in the form of DCIS and invasive cancer; ** ER—estrogen receptor; PR—progesterone receptor; HER2—human epidermal receptor 2; Luminal A—ER/PR+HER2−Ki-67 ≤ 20%; Luminal B—ER/PR+HER2−Ki-67 >20%.

**Table 4:** Causes of 86 deaths among 737 patients with DCIS, with subdivision according to the initial method of treatment of DCIS.

Cause of Deaths	Number of Deaths	Initial Method of DCIS Treatment		
		Mastectomy	Breast-Conserving Treatment	Breast-Conserving Surgery
Total number of deaths	86	47	22	17
Deaths due to DCIS recurrence	4/737 (0.5%)	3	1	0
Deaths due to other neoplastic lesions	26	11	7	8
Endometrial cancer	5	2	2	1
Lung cancer	4	1	1	2
Ovarian cancer	3	2	1	0
Contralateral breast cancer	3	2	0	1
Gallbladder/liver hilum cancer	3	1	1	1
Pancreatic cancer	2	1	1	0
Colon cancer	2	1	1	0
Gastric cancer	1	0	0	1
Cervical cancer	1	0	0	1
Melanoma	1	0	0	1
Glioblastoma multiforme	1	1	0	0
Deaths due to other causes	48	29	12	7
Missing data	8	4	2	2

## Data Availability

The data analyzed during the current study are available from the corresponding author on reasonable request.
